# Androgen dysfunction in non-alcoholic fatty liver disease: Role of sex hormone binding globulin

**DOI:** 10.3389/fendo.2022.1053709

**Published:** 2022-11-22

**Authors:** Myeong Jun Song, Jong Young Choi

**Affiliations:** Department of Internal Medicine, College of Medicine, The Catholic University of Korea, Seoul, South Korea

**Keywords:** non-alcholic fatty liver disease, insuline resistance, sexual dimorphism, androgen, sex hormone binding globhulin

## Abstract

Non-alcoholic fatty liver disease (NAFLD) is the most common form of chronic liver disease in the world. It is linked mainly to insulin resistance and metabolic syndrome including obesity and dyslipidemia. In addition, various endocrine dysfunctions including polycystic ovary syndrome (PCOS) and hypogonadism are involved in the development and progression of NAFLD. We need to know the disease pathophysiology more accurately due to the heterogeneity of clinical presentation of fatty liver disease. The liver is the major metabolic organ with sexual dimorphism. Sexual dimorphism is associated not only with behavioral differences between men and women, but also with physiological differences reflected in liver metabolism. In men, normal androgen levels prevent hepatic fat accumulation, whereas androgen deficiency induce hepatic steatosis. In women, higher androgens can increase the risk of NAFLD in PCOS. Sex hormone binding globulin (SHBG) is involved in androgen regulation. Recently, SHBG may be reported as a surrogate marker for NAFLD. Therefore, this review will focus on the mechanism of androgen dysfunction in the regulation of hepatic metabolism, the risk of developing NAFLD, and the potential role of SHBG in the course of NAFLD.; Keywords: Non-alcoholic fatty liver disease, insulin resistance, sexual dimorphism, androgen, sex hormone binding globulin

## Introduction

Nonalcoholic fatty liver disease (NAFLD) imposes a major public health burden with increasing incidence of comorbidities including obesity and dyslipidemia (1). The main histologic and imaging characteristic of NAFLD is the hepatic accumulation of lipids. Dysregulation of lipid metabolism in liver leads to the accumulation of toxic lipids, which may result in inflammation, hepatocellular injury, and fibrosis (2–4).

Recently, it was suggested that metabolic dysfunction associated fatty liver disease, which is not merely a differential diagnosis for NAFLD could reflect the current knowledge of the disease pathophysiology and aid risk stratification and management of fatty liver disease, which shows a heterogenous clinical presentation (5).

Obesity and metabolic syndrome lead to the development of NAFLD and may be accompanied by endocrine and hormonal disturbances. The liver is the major metabolic organ related to sexual dimorphism (6, 7). NAFLD prevalence is 2.0-3.5-fold higher in men than in women (8). Epidemiological studies have reported that NAFLD is also more severe in men, indicating a deleterious effect of androgens and, on the other hand, protective effect of estrogens in the pathogenesis of NAFLD (9–11). However, the sex-specific mechanisms underlying the development and progression of NAFLD remain to be elucidated.

Sex-differences in the prevalence, progression, outcomes and comorbidities of fatty liver might be considered as the result of gender differences indicative of the liver phenotype between males and females (8). The sexual dimorphism usually observed in NAFLD is reflected in polycystic ovary syndrome (PCOS) in women and hypogonadism in men. Furthermore, sex hormone binding globulin (SHBG) is related to androgen regulation (12). Recently SHBG is reported as a surrogate marker for NAFLD. Investigating the role of androgens in the development and progression of NAFLD in consideration of the gender differences is necessary. Therefore, this review will focus on the mechanism of androgen dysfunction in the regulation of hepatic metabolism, the risk of developing NAFLD, and the potential role of SHBG in the course of NAFLD.

## Hepatic lipid metabolism in NAFLD

Hepatic fatty acids (FAs) are derived from two main sources including excess carbohydrates and FAs that are produced by diet and lipolysis in the adipose tissue. Uptake of circulating lipids are facilitated by fatty acid transporters (CD36, FATP2-5) in hepatocyte membrane and is regulated by Peroxisome proliferator-activated receptor-γ (PPAR-γ) (13). Fatty acid binding protein 1 facilitate the transport of hydrophobic FAs with the cytoplasm. *De novo* lipogenesis (DNL) is regulated by the sterol regulatory element binding proteins 1c (SREBP1c) that induces the expression of genes involved in *de novo* FA synthesis including acetyl-CoA carboxylase (ACC), fatty acid synthase, the long-chain elongase and stearolyl-CoA desaturase (14). In NAFLD, enhanced SREBP1c-mediated DNL is a key character. FA oxidation is controlled by PPAR-α and reduces intrahepatic fat levels by utilizing FAs as a source of energy in mitochondrial β-oxidation. Mitochondrial dysfunction in NAFLD results in liver injury through increased production of reactive oxygen species. Triglycerides (TGs) are secreted from the hepatocytes in the form of very low density lipoprotein (VLDL) *via* the fusion of apolipoprotein B-100 (ApoB100). Decreased level of microsomal triglyceride transfer protein (MTTP) and ApoB100 may limit VLDL export and facilitate fat accumulation. An imbalance of these processes leads to the progression of steatosis to nonalcoholic steatohepatitis (NASH).

## Hepatic sexual dimorphism according to sex hormone dysfunction

Sex hormones, including estrogens and androgens play a key regulatory role in lipid metabolism and insulin sensitivity (15–17). Sex hormone dysfunction may contribute to the development of NAFLD, because a reduction in insulin sensitivity increases hepatic gluconeogenesis and lipogenesis, in turn, this may exacerbate hepatic steatosis (18).

Among the sex hormones, estrogens have critical metabolic actions in both women and men. The biologically active form of estrogens is 17β-estradiol (E2). In premenopausal women, the main source of E2 is from cholesterol, while in postmenopausal women and men, it is primarily derived from testosterone aromatization. Estrogen reversibly binds to SHBG and diffuse into liver to exert its functions by binding to estrogen receptors (ERs). Estrogen reduce TG accumulation in the liver *via* ERα. Estrogens play a key role in protecting against hepatic steatosis, by promoting lipolysis and improving FA oxidation in mitochondria *via* the induction of ACC (19). Therefore, an imbalance in estrogens may have a marked effect on hepatic lipid metabolism. Menopause, a physiological condition of estrogen deficiency, promotes the risk of development and progression of NAFLD (60% and 32% prevalence rates in menopausal and premenopausal women, respectively) (20). The incidence of NAFLD after menopause increases significantly (to that observed in men) due to the protective effect of estrogens, although gender-differences in the prevalence of NAFLD also depend on age (8). Menopause-related fat redistribution also increases the risk of insulin resistance (IR) and subsequently the risk of NAFLD (21). Accordingly, premature menopausal women are also at risk of severe liver fibrosis (22).

On the contrary, androgens showed different opposite effects by sex differences. The major circulating androgens are dehydroepiandrosterone (DHT), androstenedione, testosterone, and dihydrotestosterone, but only testosterone and DHT can bind to androgen receptors (18, 23). Testosterone plays an important role in lipid, and protein metabolism, and has a major influence on body composition including adipose fat and skeletal muscle in men (18, 24). Androgens are achieved by activation of androgen receptor (AR), followed by binding to androgen response element (ARE) or interacting with cytoplasmic signal transduction pathways, including PKA and MAPK/ERK (18). Overexpression of genes involved in lipid accumulation and down expression of FA oxidation is modulated by 5α reductase inhibition because it does not covert testosterone to DHT. Normal androgen levels prevent hepatic fat accumulation, in men, while androgen deficiency induces hepatic steatosis. In women with PCOS, higher androgen levels can increase the risk of NAFLD (25, 26). Androgen dysfunction is a significant contributor to hepatic sexual dimorphism. Therefore, determining the exact mechanisms underlying the sex dimorphism for androgens associated with the development and progression of NAFLD is necessary.

## Hyperandrogenism in women with polycystic ovary syndrome and NAFLD

PCOS affects 6–15% of women of reproductive age (27). It is characterized by chronic anovulation, hyperandrogenism (HA), and polycystic ovaries, with women often exhibiting menstrual cycle disturbances and hirsutism or acne (27). Recent studies reported a higher prevalence of NAFLD in women with PCOS than healthy controls (34-70% vs. 14-34%) (26, 28–30). In addition, NAFLD prevalence was significantly higher in patients with PCOS than healthy subjects, with an overall odds ratio of 3.93 (95% CI: 2.17-7.11) (31, 32). Conversely, women with NAFLD are diagnosed with PCOS more often than those without NAFLD (43.7% vs. 23.1%) (33). Therefore, women with PCOS should be screened for NAFLD, while premenopausal women with fatty liver should be screened for PCOS (34).

The pathophysiological mechanism that increases the risk of NAFLD in subjects with PCOS is multifactorial. IR and HA may have a bidirectional relationship with PCOS and NAFLD (35). IR represents a major physiological imbalance in patients with PCOS. As a compensatory response to IR, hyperinsulinemia develops and subsequently interacts synergistically with luteinizing hormone (LH), acting as a co-gonadotrophin within the ovary (36). The resultant activation of CYP17 promotes the production and release of androgens (37). Hyperinsulinemia also exerts extraovarian pleiotropic effects including enhancement of LH pulse amplitude, stimulation of adrenal P450c17α activity, and suppression of hepatic SHBG synthesis, thus elevating the bioavailability of free androgens (37–40).. Low SHBG levels in women can lead to progression of the characteristic phenotype of PCOS. Low SHBG concentrations are also related to NAFLD and IR in women with PCOS, and may reflect activity within a new liver-ovarian axis (41). In a recent study, two distinct PCOS subtypes were distinguished based on SHBG levels: a “reproductive” type that presents with higher SHBG levels and relatively low body mass index (BMI) and insulin levels, and a “metabolic” type that is characterized by higher BMI, glucose, and insulin levels, and lower SHBG levels (42). This finding suggests that PCOS is a heterogeneous complex disorder with different biological mechanisms (43).

HA itself is the independent risk factor to affect NAFLD, although IR is associated with high androgen levels (34). HA directly affects LDL receptors in the liver, causing an increase in LDL, that renders women with PCOS more likely to develop dyslipidemia and NAFLD (44). Furthermore, androgens may affect the production of adipokines, including leptin, and adiponectin, which could be concerned with the metabolic characteristics of PCOS and the development of NAFLD (45). In PCOS mice model, DHT increased binding of AR to ARE in elevating the SCAP-SRBEP1 interaction, resulting in increased hepatic DNL (46, 47). Chronic androgen excess induces IR and hepatic fat accumulation through mitochondrial dysfunction and causing apoptosis, and autophagy imbalance (48). Androgens can induce mitochondria β-oxidation imbalance and DNL and can exacerbate liver inflammatory injury by the overexpression of interleukin-6 (IL-6), tumor necrosis factor-α (TNF-α), MCP-1, and IL-1β (49). Therefore, HA may be a key contributor to NAFLD development in women with PCOS. The causality of the relationship between NAFLD and PCOS requires further research.

Lifestyle interventions are the most effective treatments for women with PCOS due to improvements in insulin sensitivity. Although metformin has been widely used in women with PCOS, it showed limited efficacy for resolving NASH (50, 51). A randomized clinical trial showed that liraglutide, a glucagon like pepatide-1 receptor agonist, achieved body weight loss, of 5.6%, a 66% reduction in NAFLD prevalence, and an 18% reduction in visceral adipose tissue (VAT) when administered to women with PCOS (52). However, the evidence is insufficient to recommend liraglutide as a treatment regimen in women with PCOS. Further research should aim to determine whether novel therapeutics can improve insulin sensitivity and reduce the risk of NAFLD in women with PCOS.

## Hypogonadism in men and NAFLD

Male hypogonadism is a clinical syndrome characterized by deficient or absent gonadal function that results in insufficient testosterone secretion (53). Obesity is one of the most important risk factors for secondary hypogonadism in men (54). Male obesity secondary hypogonadism (MOSH) impairs fertility, sexual function, bone mineralization, and fat metabolism, also leads to lower muscle mass and altered body composition (54). Although the prevalence of MOSH remains unclear, rates as high as 45.0–57.5% have been reported (54–56). 26% of men with NAFLD have low free testosterone in a study of 159 men randomly selected from the NASH clinical research network cohort (57). Men with low free testosterone were more likely to have NASH and advanced fibrosis than simple steatosis (88% *vs*. 67%, 27% vs. 14%, respectively) (57).

The mechanism that increases the risk of NAFLD in subjects with hypogonadism has been poorly described. Testosterone plays an important role in insulin sensitivity, body composition and lipid metabolism (58). There is a bidirectional relationship exists between low testosterone and IR (59). In preclinical study, low testosterone levels may cause hepatic fat accumulation through increased *DNL via* upregulation of hepatic SREBP-1 (60, 61). The upregulation of SREBP-2 and ACC-1 is apparently due to reduced AMP-activated protein kinase (AMPK) activity (60). Testosterone may promote the expression of hepatic scavenger receptor class B member 1, which is involved in selective uptake of cholesterol esters from circulating high-density lipoprotein (HDL) and facilitates reverse cholesterol transport. Furthermore, testosterone decreases MTTP expression, thus reducing apolipoprotein B-mediated VLDL secretion and cholesterol 7α-hydroxylase levels, which in turn leads to hepatic steatosis due to increased cholesterol uptake and decreased removal (62, 63). Testosterone also significantly increases the mRNA expression of insulin receptors, resulting in increased insulin binding, and elevated glucose oxidation (64). Serine phosphorylation of insulin receptor substrate 1, which attenuates insulin signaling by inhibiting tyrosine phosphorylation, was improved by testosterone treatment (65, 66). Testosterone deprivation leads to reduced glucose transporter type 4 expression in liver tissue and results in hyperglycemia, low insulin level, and diminished glucose uptake in adipose and skeletal muscle tissue (67).

Low testosterone and SHBG in men are independent predictors of metabolic syndrome (68). Low testosterone is related to the visceral fat distribution and exhibits sexual dimorphism due to the dependency on testosterone and E2 of men and women, respectively. Testosterone levels are inversely proportional to the amount of visceral fat. Testosterone and E2 regulate the expansion of visceral fat by activating ERs (ERα and ERβ) and ARs. ER is activated by E2 derived from testosterone aromatization; thus, testosterone deficiency, which leads to low estradiol levels, is a major cause of visceral fat deposition and IR in men (69–71). Efficient AR activation also lowers body fat and increases insulin activity (69, 72). Thus, testosterone exerts its anti-obesity effect (which inhibits the expansion of visceral fat deposition, as well as insulin and leptin resistance, leading to lipogenesis in liver and adipose tissue) by activating the AR pathway (73–75). In addition, SHBG is associated with the low testosterone levels in men with adult-onset hypogonadism (76). SHBG regulates testicular negative feedback either directly or by modulating the entry of testosterone or estradiol into cells in the hypothalamus and/or pituitary to control gonadotropin synthesis and secretion (76). Low total testosterone and SHBG were strongly associated with increased likelihood of having metabolic syndrome, independent of IR (77). Therefore, Hypogonadism is a risk factor for the development of NAFLD.

Testosterone replacement therapy in hypogonadal men with metabolic syndrome had favorable effects on hepatic steatosis, insulin sensitivity, and glucose control (78, 79). However, evidence is lacking to support the use of testosterone therapy in hypogonadal patients with NAFLD. Furthermore, the biochemical mechanisms of underlying the potential therapeutic benefits of testosterone in NAFLD remain to be elucidated, and further study is needed to understand the liver-specific role of testosterone. Studies with large cohorts are necessary to determine whether men with low androgens levels on long-term testosterone therapy are protected against prostate cancer and have a reduced risk of cardiovascular disease over time.

## Sex hormone binding globulin status in the course of NAFLD

SHBG is a glycoprotein produced by the liver (80). The primary function of SHBG is to bind and transport circulating testosterone and estradiol to regulate their bioavailability and sequester circulating androgens and estrogens (43, 81). SHGB shows high affinity for testosterone and low affinity for estradiol (81). The free testosterone in plasma is strongly influenced by the SHBG concentration because only 1-2% of testosterone in plasma is free and active; 65% is bound to SHBG and the rest to albumin. Therefore, women with low SHBG levels can have normal total testosterone levels but increased bioavailability thereof, which leads to the progression of PCOS. In addition, IR subsequently results in reproductive dysfunction by diminishing SHGB synthesis. PCOS patients with NAFLD usually have lower SHBG levels and a higher free androgen index compared with those without NAFLD, although differences in circulating androgens are not apparent (26, 34, 82, 83). Furthermore, experimental studies indicated that sex hormones bound to SHBG may directly mediate cell surface signaling, cellular delivery, and the biologic action of sex hormones (84–88). Future research on the association between the biological activity of SHBG binding fractions and risk of NAFLD is warranted.

SHBG also acts as a signal transduction factor. An experimental study showed that thyroid and estrogenic hormones increase SHBG synthesis by upregulating the expression of hepatocyte nuclear factor-4α (HNF-4α), which performs as a key factor in regulating SHBG promoter activity in the liver (89, 90). Conversely, (PPAR-γ competes with HNF-4α for a binding site on the SHGB promoter, such that PPAR-γ inhibits SHBG expression (91). SHBG levels are inversely correlated with hepatic TG and ACC activity (92). SHBG may downregulate the phosphatidylinositol 3−kinase (PI3K)/protein kinase B (AKT) pathway, which is involved in the development of local and systemic IR (93). Increased hepatic lipogenesis or IR downregulates HNF-4α expression thereby diminishing hepatic SHBG synthesis and production. In addition, inflammatory status affects the expression of SHBG. During chronic inflammation diseases, patients show increased expression of inflammatory cytokines such as IL-1 and TNF-α that affect the production of SHBG. The action of IL-1 is mediated by the NF-κB factor which downregulate HNF-4 α transcription leading to the suppression of SHGB synthesis (94).

Low testosterone is associated with a suboptimal distribution of body fat and adipocyte IR, which impairs suppression of lipolysis, and leads to ectopic fat deposition and “lipotoxicity” (95). Adipose inflammatory cytokines, such as TNF-α, IL- 6, and C-reactive protein, can impair hepatic insulin signaling and promote hepatic fat accumulation, leading to inhibition of HNF-4α mRNA *via* the activation of NF-κB or activating the Methyl ethyl ketone-1/2 (MET-1/2) and c-Jun N-terminal kinase (JNK) mitogen-activated protein kinase (MAPK) pathways (96, 97). In contrast, adiponectin increases SHBG production by activating AMPK, which increases FA oxidation and HNF-4α levels (98). Therefore, low circulating SHBG is associated with a high risk of NAFLD in men with hypogonadism.

SHBG may serve as a biomarker of NAFLD. Low testosterone and SHBG concentrations are related with metabolic syndrome and fatty liver. In a recent meta-analysis, low total testosterone was positively associated with NAFLD in men but inversely in women, on the other hand, low SHBG concentration was reportedly associated with a high risk of development of NAFLD in both men and women (99). Furthermore, SHBG has anti-inflammatory and lipolytic effects on adipocytes and macrophages, which could explain its association with lower incidence rates of metabolic syndrome and its complications (100). In a biopsy proven NAFLD study, lower SHBG levels were inversely related to the severity of steatosis (101). Therefore, improving SHBG expression can be a potential therapeutic target for NAFLD.

## Conclusion

**Figure 1:**
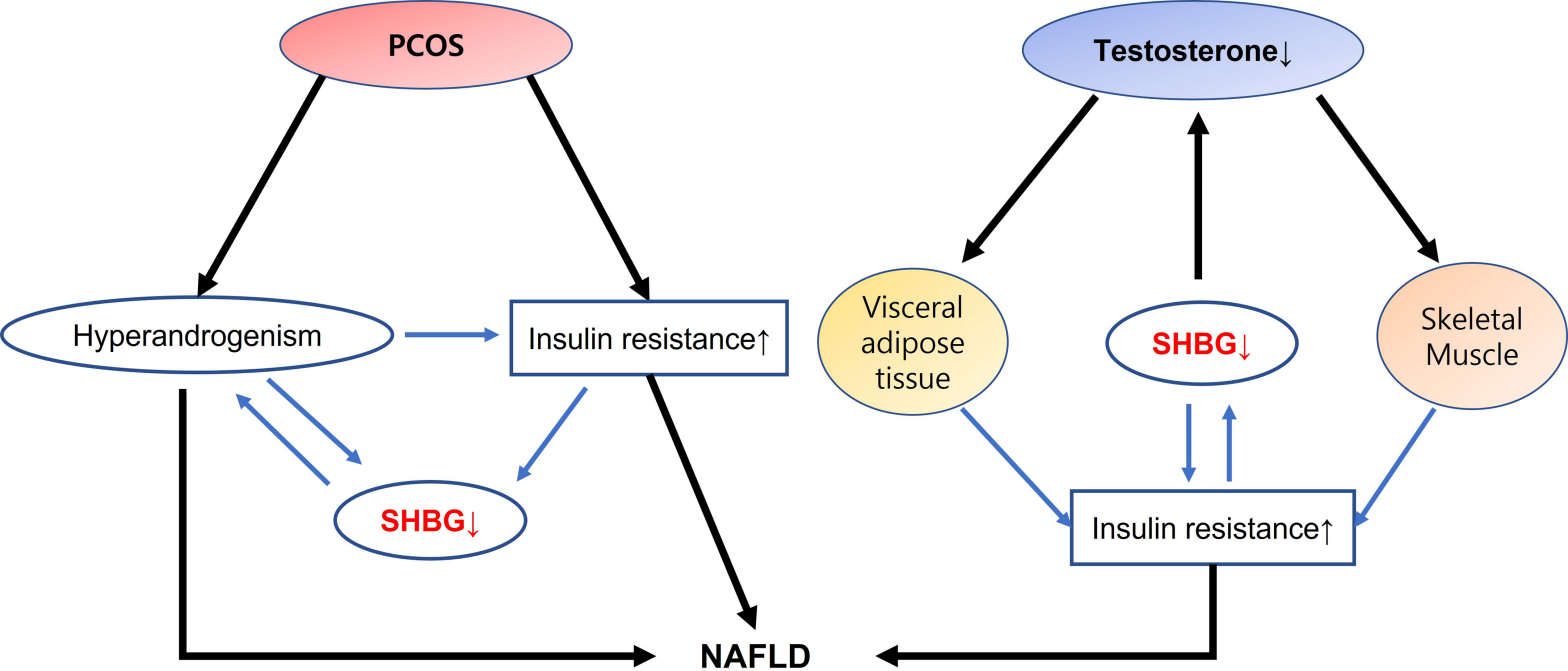
Interactions of SHBG with androgen dysfunction on NAFLD development In PCOS, HA and insulin resistance leads to downregulate SHBG production, and develops NAFLD. On the contrary, interaction of low testosterone with visceral adipose tissue and skeletal muscle may modulate insulin resistance that decreases SHBG expression and increase the risk of NAFLD. SHBG has a dual action in interacting insulin resistance as well as in acting as a buffer in the context of androgen homeostasis suggesting a role as a surrogate marker in the development of NAFLD. SHBG, sex hormone binding globulin; NAFLD, nonalcoholic fatty liver disease; PCOS, polycystic ovary syndrome; HA, hyperandrogenism.

An intricate relationship exists between androgens and NAFLD that may independently affect hepatic homeostasis. Gender differences in the effects of androgen have been observed, with low testosterone levels affecting liver function in men but not in young women, in whom HA presents a risk factor for NAFLD. An apparent bidirectional connection exists between sexual dimorphism of androgens and NAFLD. In addition, SHBGs participate in hormone regulation, acting as a buffer in the context of androgen homeostasis (**Figure 1**). Therefore, understanding the molecular mechanism of androgens in the liver would aid the development of mechanism-based therapeutic interventions for NAFLD.

## Author contributions

MJS contributed to the selection and analysis of preview literature, and wrote the original draft for this mini review. JYC supervised the manuscript and contributed to the review and editing for this mini review. All authors contributed to the article and approved the submitted version.

## Funding

This work was supported by the National Research Foundation of Korea (NRF) grant funded by the Korea government (MSIT) (No.2022R1F1A1063158).

## Conflict of interest

The authors declare that the research was conducted in the absence of any commercial or financial relationships that could be construed as a potential conflict of interest.

## Publisher’s note

All claims expressed in this article are solely those of the authors and do not necessarily represent those of their affiliated organizations, or those of the publisher, the editors and the reviewers. Any product that may be evaluated in this article, or claim that may be made by its manufacturer, is not guaranteed or endorsed by the publisher.
